# Inhibition of LDH-A by Oxamate Enhances the Efficacy of Anti-PD-1 Treatment in an NSCLC Humanized Mouse Model

**DOI:** 10.3389/fonc.2021.632364

**Published:** 2021-03-30

**Authors:** Tianyun Qiao, Yanlu Xiong, Yangbo Feng, Wenwen Guo, Yongsheng Zhou, Jinbo Zhao, Tao Jiang, Changhong Shi, Yong Han

**Affiliations:** ^1^ Department of Thoracic Surgery, Tangdu Hospital, The Fourth Military Medical University, Xi’an, China; ^2^ School of Basic Medical Sciences, Medical College of Yan’an University, Yanan, China; ^3^ Laboratory Animal Center, The Fourth Military Medical University, Xi’an, China; ^4^ Department of Thoracic Surgery, Air Force Medical Center, Beijing, China

**Keywords:** non-small cell lung cancer, NSCLC, immunotherapy, anti-PD-1, oxamate, LDH, humanized mice model

## Abstract

Immunotherapy is a curable treatment for certain cancers, but it is still only effective in a small subset of patients, partly because of the lack of sufficient immune cells in the tumor. It is reported that targeted lactate dehydrogenase (LDH) to reduce lactic acid production can promote the infiltration and activity of immune cells and turn tumors into hot tumors. Therefore, we constructed a humanized mouse model to evaluate the efficacy of using classical LDH inhibitor oxamate and pembrolizumab alone or in combination in non-small cell lung cancer (NSCLC). We found that both oxamate and pembrolizumab monotherapy significantly delayed tumor growth; moreover, combination therapy showed better results. Immunofluorescence analysis showed that oxamate treatment increased the infiltration of activated CD8+ T cells in the tumor, which might have enhanced the therapeutic effects of pembrolizumab. Treatment of the humanized mice with anti-CD8 abrogated the therapeutic effects of oxamate, indicating CD8+ T cells as the main force mediating the effect of oxamate. In conclusion, Our preclinical findings position that oxamate not only inhibits tumor growth at a high safe dose but also enhances the efficacy of pembrolizumab in Hu-PBMC-CDX mice. Our study also provides a preclinical model for exploring the efficacy of other immune-based combination therapies for NSCLC.

## Introduction

Lung cancer is a malignant tumor with the highest cancer fatality rate worldwide (1). Non-small cell lung cancer (NSCLC) accounts for 85% of all lung cancers, and most patients are found to be in advanced stages, have lost treatment opportunities, and have a low 5-year survival rate (2). In recent years, immunotherapy has brought new hope to patients. Several immune checkpoint inhibitors, such as nivolumab, pembrolizumab, atezolizumab, and durvalumab, have yielded excellent benefits in prolonging progression-free survival and overall survival for both second-line and first-line NSCLC patients, and have received US Food and Drug Administration approval (3). However, only a subgroup of patients benefits from such therapy. This is because immunotherapy is only applicable to patients with high programmed death ligand 1 (PD-L1) expression (tumor proportion score [TPS] > 50%), which is found in only a small number of patients (15%) (4). Therefore, combination therapy based on immunotherapy has become a new research direction. The purpose of combination therapy is to increase the number and activity of tumor-infiltrating lymphocytes through other treatments, thereby enhancing the effect of immunotherapy (5). Clinically, the most widely used combination therapy is chemotherapy combined with immunotherapy. In 2016, the FDA approved pembrolizumab/carboplatin/pemetrexed for first-line treatment of non-squamous NSCLC, regardless of the expression level of PD-L1. Additional treatment options include atezolizumab/carboplatin/paclitaxel/bevacizumab or atezolizumab/carboplatin/nab-paclitaxel. These combination treatments require less strict conditions than immune monotherapy (TPS > 50%). Moreover, there is no need to detect the expression level of PD-L1 and tumor mutational burden (TMB), which enables more patients to receive the treatment (4). However, in view of the strong side effects of chemotherapy both in monotherapy and combination therapy, more combination therapies with high efficacy and low toxicity need to be developed.

The Warburg effect is ubiquitous in all types of tumors, and its characteristic is that the energy metabolism of tumor cells is still dominated by glycolysis under conditions of sufficient oxygen (6, 7). Tumor cells absorb a large amount of glucose, convert pyruvate to lactic acid through lactate dehydrogenase A (LDH-A), a key enzyme in glycolysis; and oxidize nicotinamide adenine dinucleotide hydride (NADH) to nicotinamide adenine dinucleotide (NAD^+^) at the same time (8). Glycolytic metabolism in tumor cells leads to competition for limited nutrition in the tumor microenvironment and makes stromal and immune cells nutrition deficient (9, 10). Its main metabolites also affect the tumor microenvironment. A large amount of lactic acid produced by glycolysis is transported out of tumor cells through the monocarboxylate transporter (MCT), which acidifies the tumor microenvironment and promotes the secretion of vascular endothelial growth factor (VEGF) and of angiogenesis (11, 12). A large amount of lactic acid released by tumor cells can also increase the concentration of lactic acid in the tumor microenvironment and inhibit the efflux of lactic acid produced by glycolysis of T lymphocytes, resulting in acidification of the intracellular environment of T lymphocytes and inhibition of T activation, thus promoting tumor immune escape (13, 14). Therefore, as a product of glycolysis, lactic acid can not only promote tumor cell invasion, metastasis, and angiogenesis but also immune escape by acidifying the tumor microenvironment. In view of its important role in cancer metabolism, targeting lactic acid metabolism and its key enzymes is considered a potential target for cancer therapy (15). In addition, because LDH-A is not a key enzyme in normal cell metabolism, targeting it does not cause obvious symptoms, indicating that selective LDH-A inhibitors have minimal side effects. Therefore, LDH-A is considered an attractive target for the development of new anticancer drugs.

In recent years, as an inhibitor of LDH, oxamate has been proven to be a promising anticancer drug; however, its specific action mechanism is still unclear (16, 17). In previous studies, oxamate was shown to inhibit the growth and metastasis of cervical (18), gastric (19), and liver cancer cells *in vitro* (20). Furthermore, it significantly inhibited the level of LDH in mouse tumors (21). However, the traditional preclinical model is to transplant tumor cell lines or patient-derived xenografts (PDXs) into immunodeficient mice to evaluate the therapeutic effect of a drug (22). Because of the lacking human immune system, this model cannot be applied to evaluate the effect of oxamate on the immune system.

Therefore, a humanized mouse model has emerged as a promising translational platform for evaluating the efficacies of new immunotherapeutics. The human peripheral blood mononuclear cell (Hu-PBMC) mouse model or human-hematopoietic stem cell (Hu-HSC) mouse model was established by transplanting fresh PBMCs or umbilical cord blood-derived CD34+ hematopoietic stem cells into severe combined immunodeficiency mice. Then, a tumor cell line or PDX was transplanted into the mice so that the model had both the human immune system and tumor (23, 24). To date, the application of humanized mouse models has focused mainly on the evaluation of the preclinical efficacy and mechanism of new immune antibodies and is rarely used in the preclinical evaluation of combination therapy. Therefore, we believe that this is a research direction worthy of attention.

The present study aimed to investigate the potential anticancer effect of oxamate in an NSCLC humanized model and to clarify the mechanisms involved. Furthermore, this study investigated the effectiveness of oxamate in terms of potentiating the antitumor action of pembrolizumab to ascertain whether the latter can be used as an adjuvant therapy in cancer treatment.

## Materials and Methods 

### Cell Lines

H460 and H1299 cells (human non-small cell lung cancer cell lines) and HBE cells (normal lung epithelial cell line) were purchased from the Cell Bank of the Chinese Academy of Sciences (Shanghai, China). All cell lines were cultured in RPMI-1640 (Gibco) medium, supplemented with 10% heat-inactivated fetal bovine serum (FBS; Gibco) and 1% penicillin-streptomycin (Thermo Fisher Scientific, Waltham, MA, USA) at 37°C with 5% CO_2_.

### Mice

NOD-prkdc^scid^IL2rg^tm1^/Bcgen (B-NDG) mice were purchased from Biocytogen (Beijing, China) and maintained at specific pathogen-free conditions in the Laboratory Animal Center of the Air Force Military Medical University. All procedures were approved by the Institutional Animal Care and Use Committee of Air Force Military Medical University (IACUC-20200602). Mice used in these studies were provided autoclaved food and water. Mice were humanely euthanized by CO2 inhalation if a solitary subcutaneous tumor exceeded 1500 mm^3^ in area.

### Reagents

Oxamate sodium was purchased from Sigma-Aldrich Corp (St. Louis, MO, USA). Three fresh peripheral blood samples were collected from the Blood Transfusion Department of Xijing Hospital (Xi’an, Shaanxi Province, China) and approved by the Medical Ethics Committee (KY20193035).

### Establishment of Hu-PBMC Models

Human whole peripheral blood mononuclear cells were isolated using Lymphoprep (StemCell Technologies, Vancouver, BC, Canada) according to the manufacturer’s instructions. A total of 1×10^7^ human PBMCs were transplanted (ip) into 6-week-old B-NDG mice. Peripheral blood from all mice was monitored for human T cell (hCD45+hCD3+) reconstitution at the 2nd, 3rd, 4th, and 5th week. Mice that had over 25% hCD45+hCD3+ cells in the peripheral blood were considered to constitute the Hu-PBMC model. Humanized mice from different PBMC donors with different levels of hCD45+hCD3+ cells were randomly assigned to each experimental group in all experiments.

### Establishment of Immune-CDX Models

To optimally use the treatment window of humanized mice, we subcutaneously injected H1299 cells into the mice 1 week after PBMC transplantation. A total of 5×10^6^ H1299 cells in the logarithmic growth phase were collected, suspended in serum-free medium mixed with matrigel (Corning Life Sciences, Bedford, MA, USA), and injected into right flank of Hu-PBMC mice. This way, the mice were successfully constructed in the 3rd week and at the same time, the tumor reached the volume of the initial treatment (50–120 mm^3^). Body weight and tumor growth were recorded every 4 to 5 days. Tumor volume was calculated by the following formula: [length×width^2^]/2. Vehicle control saline was injected intraperitoneally every day until the study end point. In the monotherapy group, pembrolizumab (anti-PD-1; Merck, Whitehouse Station, NJ, USA) was injected intraperitoneally at a high dose (10 mg/kg) twice-weekly until the study end point. Oxamate (Sigma-Aldrich Corp) was injected intraperitoneally at 300 mg/kg every day until the study end point. The combined treatment group was simultaneously treated with pembrolizumab and oxamate until the study end point. Mice were sacrificed on day 15, and organs were removed, weighed, and processed for IHC and IFC analyses.

To deplete hCD8+ T cells in Hu-PBMC-B-NDG mice, 200 mg anti-CD8 depletion mAb (clone RPA-T8; BioLegend, San Diego, CA, USA) was injected into mice (ip) 2 days before the Oxamate treatment and then followed by weekly intraperitoneal injections of anti-CD8 mAb for 2 weeks.

### Flow Cytometry

At the completion of the study, 15 days after the start of treatment or when the total tumor burden reached 1500mm^3^ in volume, the animals were euthanized with carbon dioxide, and tissues were collected under sterile conditions. Blood was collected from the heart soon after euthanasia. The spleen and bone marrow were collected and digested into single-cell suspensions. Single-cell suspensions were incubated with fluorescently-labeled antibodies to evaluate anti-human CD45-FITC (clone HI30) and anti-human CD3-phycoerythrin (clone UCHT1). All antibodies were purchased from BD Biosciences (San Jose, CA, USA). FC500 (Beckman Coulter, Miami, FL. USA) was used for cell acquisition, and data analysis was carried out using the FlowJo software (TreeStar, San Carlos, CA, USA).

### Histology and Immunohistochemistry

Tissues harvested from humanized and non-humanized B-NDG mice were fixed with 4% paraformaldehyde for 24 h and then paraffin-embedded. The paraffin-embedded tissues were cut in 4-µm sections and were subjected to standard hematoxylin and eosin staining or immunohistochemistry (IHC). The IHC was performed with the indicated antibodies: anti-human PD-L1 (ab205921, Abcam, Cambridge, MA, USA), anti-human CD45 (ab8216, Abcam), anti-human CD4 (ab183685, Abcam), anti-human CD8 (ab108343, Abcam), and anti-human Foxp3 (ab20034, Abcam). Anti-human CD4 (EP204) antibodies were provided by Cell Signaling Technology (Danvers, MA, USA). Histology slides were scanned with the Aperio imaging system (Leica Biosystems, USA) and analyzed using the ImageScope software (Leica Biosystems).

### Immunofluorescence Microscopy

Resected tumor tissues and the spleen were fixed in 4% paraformaldehyde and embedded either in paraffin or in optimal cutting temperature (Sakura Finetek, Torrance, CA, USA) compound and frozen. Sections were deparaffinized, rehydrated, and boiled in a microwave for 20 min in 10 mM citrate buffer for antigen retrieval. Immunohistochemistry was performed using anti-human CD45 (ab40763; Abcam), anti-human CD8 (ab108343; Abcam), anti-human Granzyme B (ab208586; Abcam), anti-human PD-1 (ab234444; Abcam) and anti-human TNF-alpha (ab215188; Abcam). The secondary antibody included goat anti-rabbit IgG AF488 (ab150077, Abcam) and goat anti-mouse IgG AF647 (ab150115, Abcam). Sections were incubated overnight at 4°C before being incubated with the appropriate Alexa Fluor-conjugated secondary antibodies. Slides were washed between staining steps with Bond Wash (Leica) and stripped between each round of staining with heat treatment in antigen retrieval buffer. Nuclei were counterstained with DAPI (ab104139, Abcam). For acquisition, data were acquired by sequential acquisition, and tile-scan imaging was performed on an SP8 confocal microscope (Leica Microsystems, Wetzlar, Germany).

### Detection of Cell Viability and LDH Activity

The cell counting kit-8 (CCK-8) assay kit (Dojindo, Shanghai, China) was used to test the effects of oxamate sodium on cell viability at different concentrations or times. Cells were seeded at 5×10^3^/well and treated with different concentrations of oxamate sodium (0-100 mmol/l) for 12, 24 and 48 h, respectively. Ten microliters of CCK-8 solution was added into each well, then incubated in the dark for another 3 h. Optical density was measured using a microplate reader (Bio-Tek Instruments, Inc., Winooski, VT, USA) at 450 nm.

Intracellular LDH activity was determined using an LDH activity assay kit (Solarbio, Beijing, China). Cells treated with different concentrations of oxamate were harvested and lysed for protein to measure the LDH activity. As for glycolysis stress test, Seahorse Bioscience cell energy metabolism analysis system was used. Cells were plated on Cell-Tak-coated Seahorse culture plates (50,000 cells per well) in medium consisting of minimal, unbuffered DMEM supplemented with 2 mM glutamine. Basal rates were taken for about 25 min. Cells were stimulated with 10 mM glucose, 2 μM oligomycin and 10 mM 2-DG.

### Statistical Analysis

All data are expressed as the mean ± standard error (SE). Statistical analyses of all data were performed by GraphPad Prism (Version 7.0, GraphPad Software, Inc, San Diego, CA, USA). Differences between groups were tested with analysis of variance, and p < 0.05 was considered significant.

## Results

### Inhibition of LDH by Oxamate Suppresses Cell Viability in NSCLC Cells

**Figure 1:**
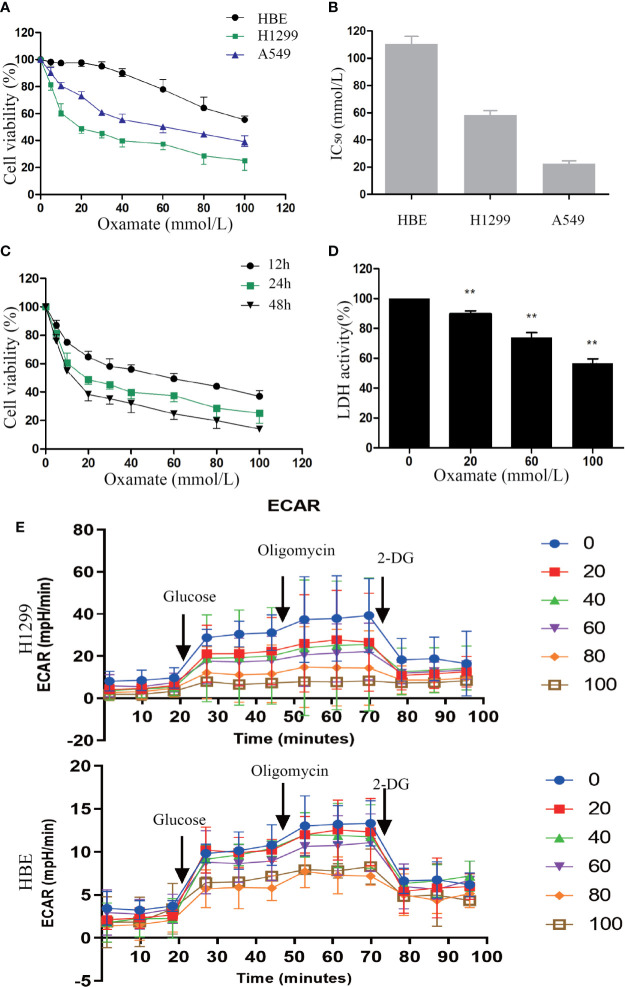
Different effects of oxamate on the cell viability and LDH enzyme activity in NSCLC cells and normal lung epithelial cells. **(A)** A549, H1299 and HBE cells were exposed to varying doses of oxamate for 24 h, and tested by CCK-8 assay. Cell viability was calculated as a percentage of untreated cells (100%); values are represented as means ± SD; n=4. **(B)** IC_50_ of different cells at 24h were calculated by the growth curves. **(C)** The effect of oxamate on H1299 cells was determined at different time points. **(D)** Cells were treated with 0, 20, 60 and 100 mM oxamate for 24 h and using a commercially available kit to determine the intracellular LDH enzyme activity. Values are represented as means ± SD; n=6. **P<0.01 vs. the untreated control. **(E)** Cells were treated with 0, 20, 40, 60, 80 and 100 mM oxamate for 24 h and using a glycolysis stress test to determine the glycolysis ability. 2-DG, 2-deoxy-d-glucose; oligo, oligomycin; Values are represented as means ± SD; n=4.

First, to explore the effect of oxamate on cell proliferation in NSCLC cells, CCK-8 (cell counting kit-8) assay were conducted. H1299, A549 cancer cells, and normal lung human bronchial epithelial (HBE) cells were treated with different doses of oxamate for 24 h. We observed that oxamate significantly inhibited the viability of H1299 and A549 cells in a dose-dependent manner, while it had little effect on HBE cells (**Figure 1A**). The IC_50_ (50% inhibitory concentration) values of oxamate for H1299 and A549 cells were 32.13 ± 2.50 and 19.67 ± 1.53 mmol/L, respectively, but for HBE cells, the value was very high at 96.73 ± 7.60 mmol/L (**Figure 1B**). Next, given its high sensitivity to oxamate, we selected H1299 cells and treated them with different doses of oxamate for 12, 24, and 48 h, and found that the inhibition of H1299 by oxamate was also time-dependent (**Figure 1C**). These results indicate that oxamate has an obvious killing effect on tumor cells but has low cytotoxicity on normal human cells. This provides a basis for the application of oxamate for inhibiting the production of lactic acid *in vivo*.

In order to further determine the previously reported inhibitory effect of oxamate on LDH, we used commercial kits to detect the activity of LDH in cells treated with different doses of oxamate. Oxamate could reduce the LDH activity of H1299 in a dose-dependent manner, thus reducing the production of lactic acid (**Figure 1D**). Furthermore, we also examined the effects of different concentrations of Oxamate on glycolysis of H1299 and HBE cells. The results showed that 24-hour Oxamate treatment could significantly inhibit the glycolysis activity of tumor cells in a dose-dependent manner while have limited effect on HBE cells(**Figure 1E**). The result demonstrated that LDH inhibition by oxamate disturbed glycolysis and decreased lactic acid production in the NSCLC cells.

### Establishment and Identification of a Humanized Mouse Model

**Figure 2:**
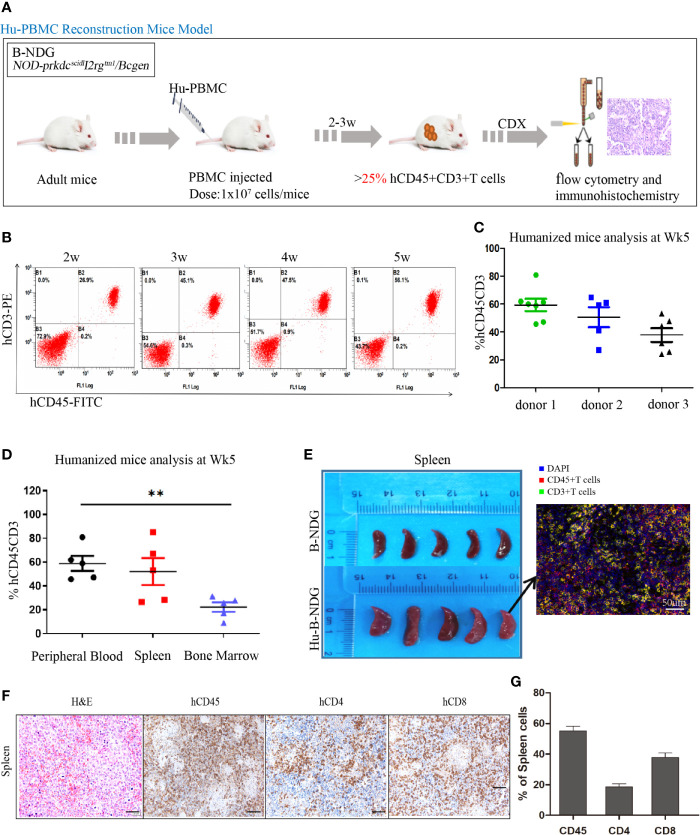
Construction and identification of humanized mouse model of immune system. **(A)** Adult B-NDG mice were injected with 1 × 10^7^ PBMC (iv), followed by subcutaneous injection of 5 × 10^6^ tumor cell lines. 2-3 weeks later, the peripheral blood of mice was collected from the tail vein to detect the content of hCD45+CD8+T cells, which marked the successful construction of the Hu-PBMC-CDX mouse model when it was more than 25%. **(B)** Dynamic flow detection of hCD45+CD3+T cell level in peripheral blood of a representative Hu-PBMC mouse model. **(C)** Reconstruction level of humanized mice constructed by PBMC from three different donors at the fifth week, n=5. **(D)** Reconstruction levels of hCD45+CD3+ T cells in peripheral blood, spleen and bone marrow of humanized mice at the 5th week, n=5. **P<0.01. **(E)** The spleen of normal B-NDG mice was compared with that of Hu-PBMC mouse model mice, n=5. **(F, G)** Immunohistochemistry for CD45, CD8, CD4, and H&E stains in spleen are shown. Representative images were taken at 20x magnification and the scale bar denotes 50 mm.

The construction strategy of the humanized mouse model is shown in **Figure 2A**. The isolated PBMCs were injected into adult mice, and the reconstitution level of human T (hCD45+CD3+) cells in the peripheral blood of the mice was dynamically monitored using flow cytometry (**Figure 2B**). With the extension of reconstruction time, human T (CD45+CD3+) cells increased significantly. We also observed that there were differences in the level of human T cells among different donors. As shown in **Figure 2C**, at the 5th week, the reconstruction levels of donor 1 and donor 2 were better than that of donor 3; therefore, in the follow-up experiment, we used the frozen PBMCs of donor 1 and donor 2 to construct the humanized mouse model. After successful reconstruction, the humanized mice reconstructed from two donor sources were randomly assigned to four experimental groups. Humanized mice generally undergo graft-versus-host reaction (GVHD) at the 5-6th week of reconstruction, including weight loss, arched back, hair removal, and other GVHD reactions.

In addition, the reconstruction levels of different organs in humanized mice are different, and we focused on immune organs such as the spleen and bone marrow. The results showed that the level of human T cells in the peripheral blood was similar to that in the spleen but significantly higher than that in the bone marrow (**Figure 2D**). We also observed that the spleen of humanized mice was larger than that of normal mice, which may be caused by an infiltration of a large number of human immune cells (**Figure 2E**). To further determine the immune subpopulations in spleen, we performed an immunohistochemical analysis and found that CD45+ cells showed high infiltration in the spleen, along with high infiltration of CD4+ and CD8+T cells (**Figures 2F, G**). These results indicated that the humanized mice were reconstructed successfully, and all organs were infiltrated with immune cells.

### Hu-PBMC-B-NDG Mice Support Tumor Growth of NSCLC Cell Line

**Figure 3:**
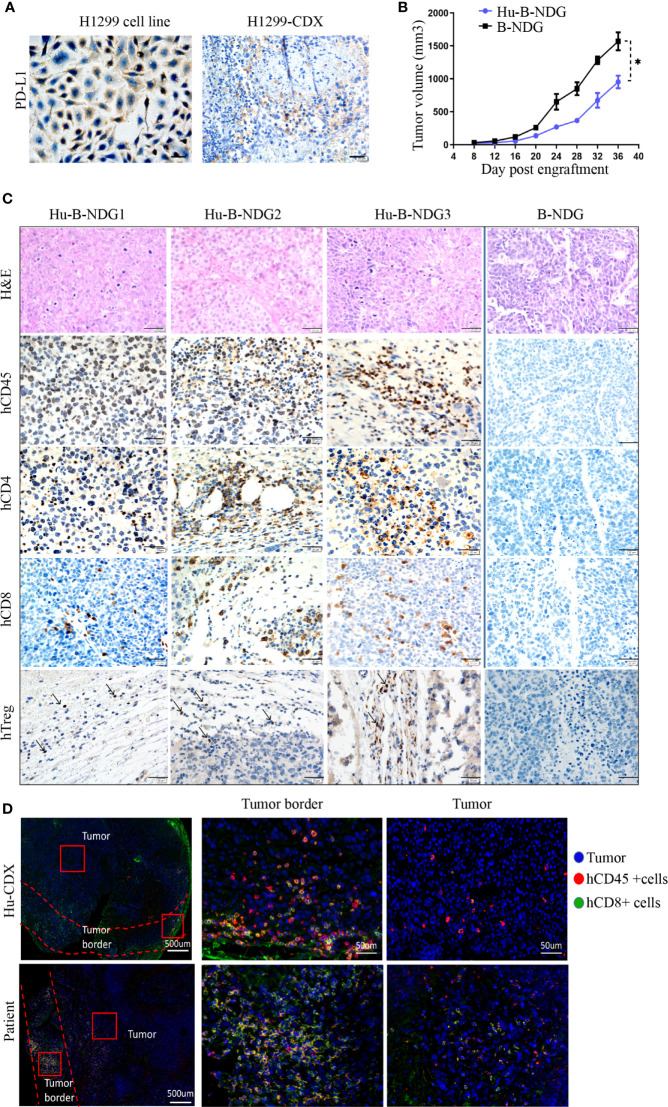
Growth of NSCLC CDX in Hu-PBMC-B-NDG mice. **(A)** Immunohistochemical analysis of PD-L1 expression in H1299 cell line and CDX. **(B)** Growth curve of H1299 cell line on B-NDG and Hu-PBMC-B-NDG mice.Data are presented as the mean ± SD of the volume of CDX (mm^3^); n = 5. *P<0.05. **(C)** Immunohistochemistry for CD45, CD8, CD4,Treg(Foxp3) and H&E stains in three tumours from humanzied B-NDG mice and one tumour from B-NDG mice are shown. Representative images were taken at 40x magnification and the scale bar denotes 20 mm. **(D)** Immunofluorescence was used to analyze the immunocyte infiltration in the center and border of tumors from Hu-PBMC-CDX and clinical patients. Representative images were taken at 40x magnification and the scale bar denotes 50 mm.

We analyzed the expression of PD-L1 in the H1299 cell line and found that its PD-L1 was moderately expressed. To confirm that high PD-L1 expression continues after tumor formation, we transplanted the H1299 cell line into B-NDG mice to construct a CDX (cell-line-derived xenograft) model, and found medium PD-L1 expression in the tumors (**Figure 3A**). In order to determine whether the human immune system has an effect on tumor growth, we transplanted H1299 cell lines on non-humanized mice and compared the tumor growth rate between non-humanized and humanized mice. The growth of tumor cell lines in humanized mice was slower than that in non-humanized mice, indicating that the immune system had a certain retarding effect on tumor growth (**Figure 3B**). Moreover, we performed hematoxylin and eosin staining and histochemical analysis of the tumors and found CD45+, CD8+, CD4+, and Treg immune cell infiltrations in the tumors grown in humanized mice. These results suggest that the NSCLC cell line can grow in humanized mice and have immune infiltration (**Figure 3C**).

For understanding the characteristics of immune infiltration in tumor tissue, we further analyzed the infiltration of CD45+ immune cells and CD8+ T cells in the tumor using immunofluorescence. We observed that CD45+ immune cells mainly gathered at the edge of the tumor, while the immune infiltration in the center of the tumor was very low, which was similar to the tumor immune cell infiltration in clinical patients (**Figure 3D**). We believe that the low immune infiltration in the center of the tumor in CDX and clinical specimens may be the main reason for the limitation of tumor immunotherapy. These results signify that the Hu-PBMC-CDX model can simulate clinical tumor infiltration to a certain extent and is an ideal preclinical model for immunotherapy.

### Evaluating the Antitumor Effect of Oxamate and Anti-PD-1 Monoclonal Antibody in Hu-PBMCs-CDX Mouse Model

**Figure 4:**
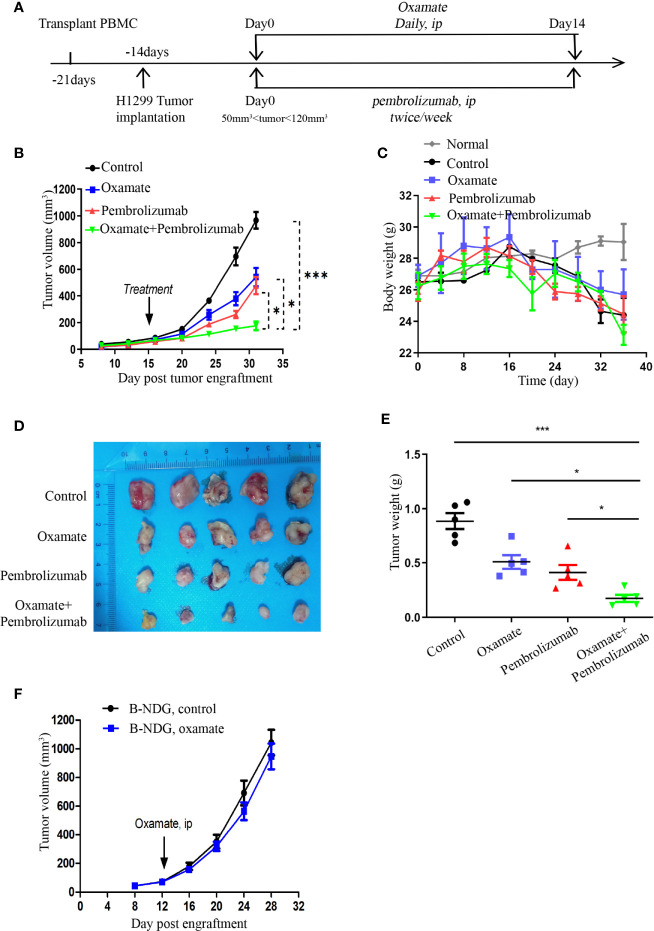
Effects of Oxamate alone or in Combinations with Pembrolizumab on NSCLC Hu-PBMC-CDX. **(A)** Experimental timeline for treatment NSCLC CDX with Oxamate and pembrolizumab in Hu-PBMC-B-NDG mice. **(B)** Tumor growth curve of control group, Oxamate monotherapy group, Pembrolizumab monotherapy group and combination treatment group. Data are presented as the mean ± SD of the volume of CDX (mm^3^); n = 5. *P<0.05, ***P<0.001. **(C)** The body weight of mice in each treatment group and normal mice without PBMCs transplantation was monitored every four days from the beginning of PBMCs transplantation to the end of treatment; n=5. **(D, E)** At the end of treatment, the tumors of each treatment group were collected, photographed and weighed; n = 5. *P<0.05, ***P<0.001 **(F)** Tumour growth after treated with Oxamate or PBS in B-NDG mice bearing NSCLC cell line. Group mean differences between Oxamate vs. controls were not significant; n = 5.

When the volume of Hu-PBMCs-CDX tumor reached 50–120 mm^3^, the treatment was commenced and the treatment scheme is illustrated in **Figure 4A**. Daily oxamate treatment can significantly slow down tumor growth; moreover, pembrolizumab monotherapy also inhibits tumor growth. Notably, the effect of the combination therapy was better than that of the two separate monotherapies, indicating that oxamate enhances the therapeutic effect of pembrolizumab (**Figure 4B**). For evaluating the safety of drug treatment, we dynamically monitored the body weight of mice in each treatment group. The results indicate that despite the daily high dose, there was no abnormal weight loss in the oxamate monotherapy group compared with the control group, which corresponds to the aforementioned low toxicity of oxamate to human HBE cells, indicating that it has a large safety range. At the same time, no significant weight loss was observed in the mice in the combined treatment group, indicating that the combined treatment did not increase toxicity (**Figure 4C**). Fifteen days after the start of treatment, the mice were euthanized, and the tumor tissues were collected. As shown in **Figures 4D, E**, the tumor volume and weight of the combined treatment group were significantly reduced, showing a good therapeutic effect.

In addition, to investigate whether the underlying mechanism of tumor growth inhibition by oxamate is mediated by the immune system, we treated the unreconstructed tumor-bearing mice with oxamate and found that the tumor growth was only slightly delayed compared to that in the control mice, but the results were not statistically significant (**Figure 4F**). This indicates that the inhibition of tumor growth by oxamate is mediated by the immune system, which inspired us to explore the underlying mechanism of oxamate promoting immunotherapy.

### Evaluating the TIL After Treatment in Hu-PBMCs-CDX Mouse Model

**Figure 5:**
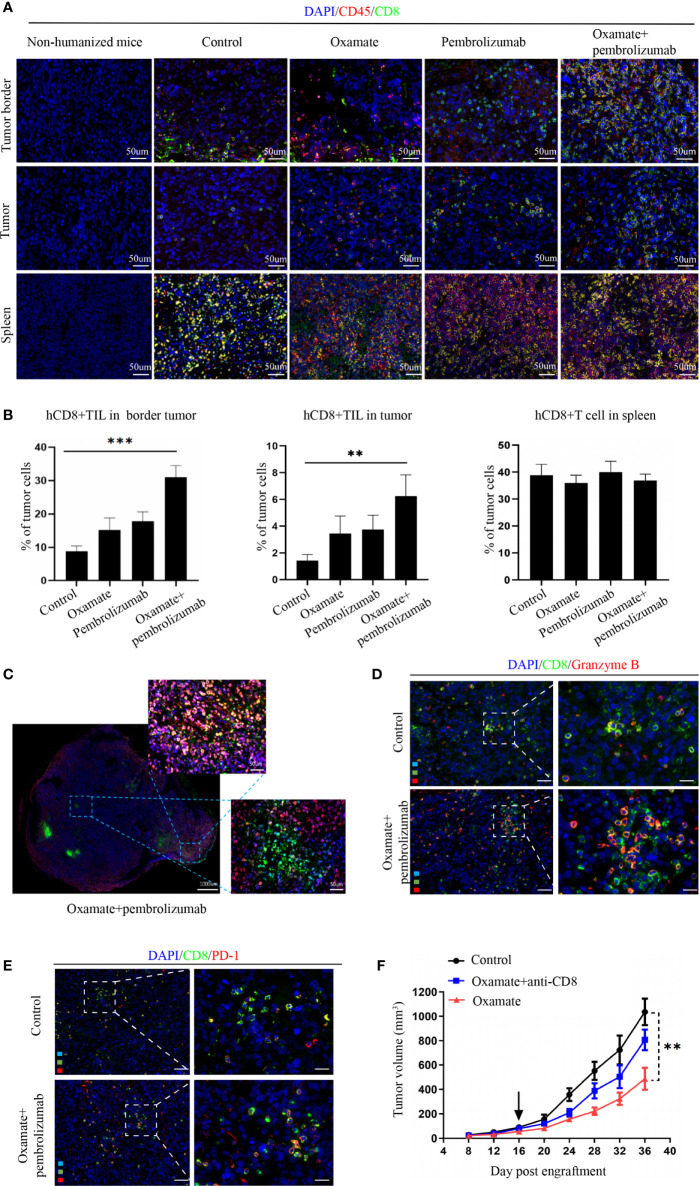
Analysis of tumor immune cell infiltration in NSCLC Hu-PBMC-B-NDG mice after treatment. **(A)** The infiltration of CD45+ and CD8+ immuno cells in the spleen and tumours in each treatment group was analyzed by immunofluorescence. CD45+(Red) CD8+(Green). **(B)** Immunofluorescence quantitative analysis of CD8+T cells in tumor and spleen of each treatment group. **P<0.01, ***P<0.001. **(C)** Immunofluorescence of a representative CDX after treated with oxamate + pembrolizumab. **(D)(E)** Immunofluorescence was used to analyze the activation of CD8+ cells in the control group and the combined treatment group, and CD8+ Granzyme B + T cells and CD8+ PD-1+ T cells were labeled respectively. **(F)** Tumour growth curve of Hu-PBMC-CDX treated with oxamate alone or oxamate plus anti-CD8 depletion mAb. Data are presented as the mean ± SD of the volume of CDX (mm^3^); n = 6. **P<0.01.

For studying the underlying mechanism of immunotherapy, we performed immunofluorescence analysis on the tumor tissues of each treatment group to observe the changes in immune cell infiltration after treatment. Previous studies have reported that CD8+ cytotoxic T cells mainly mediate the effect of tumor immunotherapy, so we analyzed the number of CD8+ cells in different parts of the tumor. In the periphery of tumors, oxamate alone increased CD8+ immune cells, whereas the combination with pembrolizumab resulted in a significantly higher proportion compared with that of either treatment alone or control. In the center of tumors, CD8+ immune cells increased with the combined regimen compared with that of either pembrolizumab or oxamate. No statistical difference was found between oxamate and pembrolizumab, both in the center and periphery of tumors (**Figures 5A, B**). This finding suggests that increased infiltration of CD8+T cells both in the periphery and center might have caused the therapeutic effect of oxamate. We also compared the immune infiltration of the spleen in each treatment group, and found no significant difference among the groups, indicating that the effect of each treatment group on immune cells was limited to the tumor site. As shown in **Figure 5C**, CD8+ T cell infiltration in the center of the tumor increased after combined therapy. Furthermore, in order to analyze the activation state of increased CD8+ T cells in the combined treatment group, we performed immunofluorescence staining of CD8+ Granzyme B + and CD8+ PD-1+ in the control group and the combined treatment group. The results showed that the proportion of activated CD8+ cells in the combined treatment group increased significantly. This shows that combined therapy can not only increase the infiltration of CD8+ cells, but also change its immunosuppressive state (**Figures 5D, E**).

Further, to verify the role of CD8+T cells in oxamate treatment, we used CD8+ antibodies in the reconstructed humanized mice to remove CD8+ immune cells from the mice, followed by oxamate treatment to observe the effect (**Figure 5F**). Compared with normal humanized mice, oxamate had no inhibitory effect on tumor growth in Hu-PBMCs-CDX mice treated with the CD8 antibody. This shows that the effect of oxamate is mainly mediated by CD8+T cells. One of the possible mechanisms by which oxamate could enhance the effect of immunotherapy is by increasing the infiltration of CD8+T cells in the center of the tumor by reducing the production of lactic acid.

## Discussion

In this study, we found that oxamate effectively inhibited the activity of LDH at the cellular level, thus reducing the production of lactic acid. In fact, in view of the inhibitory effect of a highly acidic environment on the immune system, researchers have been investigating ways to inhibit the tumor glycolysis pathway, such as targeting key enzymes of glycolysis [FX-11 (25) and oxamate (26)], lactic acid excretion [MCT-1/MCT-2 inhibitor like diclofenac (27)], and proton pump inhibitors [esomeprazole (28)], to combine them with immunotherapy. In one study, esomeprazole combined with adoptive cell transfer (ACT), a therapy based on the transfer of tumor reactive immune cells, increased the survival of mice loaded with B16 melanoma (29). Previous studies have reported that oxamate can inhibit LDH-A activity and CDX growth in nasopharyngeal carcinoma (30), and breast cancer (21) meanwhile enhances their sensitivity to radiotherapy or chemotherapy. However, the combined application of oxamate and immunotherapy has not been explored. Our study showed that oxamate can be effectively combined with immunotherapy to enhance its anti-tumor effect, and it has minor side effects on normal cells and tissues despite its high dose. In addition, unlike proton pump inhibitors (31), LDH-A is not a necessary enzyme for normal cell metabolism, so the overall effect of its inhibition by oxamate on the human body is very small, which is also conducive for its future clinical application.

In this study, we constructed the Hu-PBMC-B-NDG model and conducted the first preclinical combined therapy trial for NSCLC, which proved that the therapeutic effect of oxamate combined with pembrolizumab was better than that of a single drug. Previous studies have reported that in immune-intact mouse models, oral alkaline buffer or transplantation of LDH-A-deficient tumors to inhibit the acidic microenvironment of tumors can enhance the therapeutic effect of anti-PD-1 and improve the immunosuppressive state of the tumor microenvironment (32, 33). These studies show that it is feasible to target lactic acid metabolism to enhance the effect of immunotherapy. However, these anti-PD-1 antibodies are mouse antibodies rather than clinical antibodies, such as pembrolizumab and nivolumab. Therefore, the main purpose of our study was to observe whether drugs targeting lactic acid metabolism can enhance the efficacy of anti-PD-1 antibodies that have been used clinically. With the development of immunotherapy, humanized mouse models with both the human immune system and tumor cells have become an indispensable preclinical research model for immunotherapy research. At present, most studies only evaluate the anti-tumor effect and the mechanism of antibody monotherapy in humanized mice, and there are only few preclinical trials of combined therapy (34, 35). Therefore, our study provides a good basis for the combined application of drugs targeting metabolism and the immune system in clinical practice. The humanized mouse model could also be used to study additional immune-based combination therapies, such as the combination of angiogenic factor inhibitor (VEGF Inhibitor) and anti-PD-1.

Our study showed that the increase in the immunotherapy effect of combined treatment with oxamate is mediated by increased CD8+T cell infiltration in tumor tissues. Jerome Galon and Daniela Bruni categorized the tumors into hot, altered (excluded and immunosuppressed), and cold tumors, according to the distribution of cytotoxic CD3+T cells and CD8+T cells in the tumor tissue. They proposed that the immunoscore of the tumor tissue is positively related to the effect of immunotherapy (36). Our study showed that NSCLC CDX constructed in humanized mouse models belongs to altered-excluded tumors, in which CD3+ and CD8+ T cell infiltrates are low at the tumor center and high at the margin, resulting overall in an intermediate immunoscore. Therefore, in NSCLC, the key to the success of immunotherapy-based combination therapy is to transform altered tumors into hot tumors (37, 38). In a previous study, Brand et al. injected a Ldha^low^ cell line into C57BL/6 mice and found that the number of CD8+T cells in the Ldha^low^ tumor was higher than that in the control group and was accompanied by functional activation, which may be achieved *via* the upregulation of nuclear factor of activated T cells (NFAT) (39). In our study, we found that this increase mainly took place at the center of the tumor and that the removal of CD8+T cells can eliminate the effect of immunotherapy. As for the reason why oxamate increases tumor immune infiltration, a previous study suggested that the decrease of lactic acid level in tumors can downregulate the expression of PD-L1, thus strongly blocking the PD-1/PD-L1 pathway. Leading to elevation of pro-inflammatory anti-tumor responses such as higher infiltration and activity of CD8+ cytotoxic cells and a diminished frequency of Treg cells (33). In addition, it has been reported that targeted lactic acid metabolism can reduce the production of angiogenic factor (VEGF) by downregulating hypoxia-inducible factor 1a (HIF1a), which contributes to the normalization of tumor blood vessels and increases the infiltration of immune cells (40). We believe that the immune-promoting effect of oxamate may be more complicated and go beyond mere interference with the VEGF and PD-1/PD-L1 signaling pathways. This aspect is worth exploring in-depth in future studies.

The immune system of a humanized mouse model constructed by PBMCs is mainly composed of human T cells and lacks immune subpopulations such as B cells and NK cells, so the limitation of our study is that we could not study the effect of oxamate on these subpopulations and the role of these subpopulations in immunotherapy. In a follow-up experiment, we will use the Hu-HSC-B-NDG mouse model constructed by CD34+ hematopoietic stem cells to further study the effects of oxamate on B cells, NK cells and others. In general, this study successfully created a humanized mouse model of NSCLC to study the effect of combined therapy and its mechanism. We proved that oxamate can both inhibit tumor growth and enhance the efficacy of pembrolizumab by increasing the number of immune infiltrating cells. This provides a preclinical model and a basis for follow-up experiments on combined therapy of drugs targeting metabolic pathways and immune drugs.

## Data Availability Statement

The raw data supporting the conclusions of this article will be made available by the authors, without undue reservation.

## Ethics Statement

The animal study was reviewed and approved by Institutional Animal Care and Use Committee of Air Force Military Medical University.

## Author Contributions

Q-TY contributed to the conception, design, data acquisition, and analysis and drafted and critically revised the manuscript. Y-LX, Y-BF, W-WG, Y-SZ, J-BZ, and TJ contributed to data acquisition and critically revised the manuscript. C-HS and YH contributed to conception, data analysis, and interpretation and drafted and critically revised the manuscript. All authors contributed to the article and approved the submitted version.

## Funding

The National Natural Science Foundation of China (81772462) and (81001041) provided funding support for the design, analysis, and publication of this study.

## Conflict of Interest

The authors declare that the research was conducted in the absence of any commercial or financial relationships that could be construed as a potential conflict of interest.
